# Optimization of Adsorbent Layer Type and Developing Solvent in Coccidiostats Sample Preparation with Procedure of Solvent Front Position Extraction

**DOI:** 10.3390/molecules25246011

**Published:** 2020-12-18

**Authors:** Maciej Jan Rybicki, Anna Klimek-Turek, Tadeusz Henryk Dzido

**Affiliations:** Department of Physical Chemistry, Medical University of Lublin, ul. Chodźki 4a, 20-093 Lublin, Poland; annaklimekturek@umlub.pl (A.K.-T.); tadeusz.dzido@umlub.pl (T.H.D.)

**Keywords:** sample preparation, thin-layer chromatography, Solvent Front Position Extraction, coccidiostats, liquid chromatography-tandem mass spectrometry

## Abstract

Coccidiostats are drugs used against coccidiosis, a common disease among breeding animals. Their widespread application leads to the appearance of their residues in food, which is potentially harmful for human health and life. The European Union has established limits of concentrations of these drugs in premixtures and food. Nowadays, there are many methods for monitoring coccidiostats’ presence in market products, but their frequent weakness is sample preparation. Solvent Front Position Extraction is a planar chromatography-based sample preparation method that allows for effective assay of samples with coccidiostats when coupled with LC-MS/MS. The purpose of this research was to find common conditions for the effective isolation of eight coccidiostats from biological sample components with both lower and higher retention than the substances of interest. The acquired results were used for effective isolation of monensin and salinomycin from the premixture samples and allowed for their quantitative determination. The application of a semi-automatic device for the development of chromatograms positively impacted the results, confirming the effectiveness of the method for determining coccidiostats in biological samples.

## 1. Introduction

Coccidiostats are group of veterinary antiparasite drugs against protozoa from Eimeria genus [1]. They are widely used in animal husbandry. Their use is regulated by European Union law, because their residues in meat and other animal products are potentially harmful for human health [2]. They are administered to animals as premixtures, which are defined, following Regulation 1831/2003, as “mixtures of feed additives or mixtures of one or more feed additives with feed materials or water used as carriers (…)” [3]. The maximal concentrations of these drugs in feed are established in individual regulations, and EU countries are obliged to monitor these values, because exceeding them significantly increases the probability of the contamination of food by coccidiostats.

Premixtures, as biological materials, have complex compositions, so isolation of coccidiostats from them is impeded. For this reason, there is a serious need for a sample preparation method of great effectiveness and robustness, coupled with LC-MS, which nowadays is claimed to be the most reliable method for the detection and determination of these drugs [4]. There are many sample preparation methods that fulfill the listed requirements, but all of them are characterized by many disadvantages. Moloney et al., described a method using one-step liquid extraction with acetonitrile [5]. Simplicity was undoubtedly its advantage, but the effectiveness of this method was potentially poor. Piatkowska et al. used modified SPE [6], as did Dubois et al. [7], but the described procedures were laborious. QuEChERS and its modifications have been applied for samples with coccidiostats, as in the paper by Stubbings and Bigwoods, but this procedure was complicated and applicable only to a narrow range of compounds [8]. Chico et al. presented an interesting method for cleaning up the sample with gel-permeation chromatography, but it was poorly investigated, and so it will not be available for common use any time soon [9].

A new sample preparation technique, Solvent Front Position Extraction (SFPE), has been developing as a cheap and time-saving procedure. It is based on thin-layer chromatography. It was developed as a response to the studies by Oellig and Schwack on sample preparation with planar solid phase extraction [10,11]. Liquid samples, with a previously applied internal standard, are applied on the start line of a chromatographic plate. Then, in the first stage of sample preparation, a chromatogram is developed horizontally by mobile phase solution with an elution strength lower than that required for elution of the substance(s) of interest and an internal standard with the front of the solvent. Mobile phase is distributed by movable pipette [12,13]. Matrix components with lower retention than the substance(s) of interest and the internal standard migrate longer distances than the substance(s) of interest and the internal standard. Then, the mobile phase is evaporated. In the second step of the procedure, a chromatogram is developed by mobile phase with an elution strength stronger than that used in the first stage of the procedure to achieve a distance a few mm longer than the migration distance of the least-retained substance of interest. If necessary, the second step is repeated to the point at which the substance(s) of interest and the internal standard form a narrow zone. Consequently, matrix elements with greater and lower retention are separated from the substance(s) of interest and the internal standard. In fact, the substance(s) of interest and the internal standard can be easily transferred to the LC-MS system through extraction from the position of the front of the solvent, as mentioned above, leading to its denotation as Solvent Front Position Extraction (SFPE). It is important to note that in SFPE, the zone of the substance of interest and the internal standard is usually visible without derivatization, which is undoubtedly an advantage of this technique.

One of the most recent reports presented an effective technique using a multi-componential sample with a biological matrix [14]. A semi-automatic device was also introduced for the fast and simultaneous development of chromatograms of many samples [12,13]. A previous paper described the preparation of biological sample, which included five coccidiostats. One chromatographic system was investigated that included methanol and silica gel plates, which was sufficient for obtaining satisfactory results for the determination of substances of interest by LC-MS/MS. However, the substances of interest were only separated from matrix components with higher retention than the substances of interest, while substances with lower retention were not separated. In addition, the tested biological samples were prepared by enriching animal feed and bovine serum with substances of interest, which wasn’t representative for samples acquired from market [15].

This article shows efforts and positive results towards the optimization of the SFPE procedure; it is applicable to eight coccidiostats in methanolic solutions (or DMSO when necessary). In this study, the main goal was to determine the conditions that would allow the separation of matrix substances with lower retention from the substances of interest. The newly established conditions, sought among a broad range of combinations of stationary phase and mobile phase, were successfully applied for the effective isolation of coccidiostats from two commonly available premixtures on the market (constituting biological samples) and their quantitative determination. Furthermore, a semi-automatic device was also used for the first time for the sample preparation of these premixtures. The mode of action of this instrument has been described in detail before, so in this paper, there is no need for repetition.

## 2. Results and Discussion

### 2.1. Investigation of Various Systems of Stationary and Mobile Phases

An important advantage to the SFPE sample preparation procedure is the possibility of separating the substances of interest from the matrix components with both higher and lower adsorption energy than the substance of interest and the internal standard (look: Introduction). For this reason, mobile phases that allow substances of interest to achieve values of retardation factor of about 0.5 after the first step of the procedure were searched for. Therefore, the study began with determining the relation between the retention of coccidiostats and the combination of the stationary and mobile phase. The most common stationary phases of various properties were investigated for the development of coccidiostats: HPTLC Cellulose F, HPTLC CN F_254_, HPTLC Diol F_254_, HPTLC RP-18 F_254_ and HPTLC Silica gel 60 F_254_. As mobile phases, toluene, methanol, acetone, heptane and acetonitrile were used.

It was expected that a large number of available combinations of stationary and mobile phases would result in the establishment of at least a few effective chromatographic systems. The obtained results are presented in Table 1.

On the basis of the data presented in Table 1, it can be noted that systems with methanol, acetone and acetonitrile had very high retardation factor values for all substances, often close to or equal to 1. In turn, in systems with heptane and toluene, coccidiostats usually showed very high retention. Consequently, mixing appropriate volumes of solvents with relatively high elution strength (acetone, methanol and acetonitrile) with solvents with low elution strength (toluene, heptane) could result in obtaining a mobile phase with sufficient elution strength for separation of the matrix components with lower and higher adsorption energy from the substances of interest.

There were some interesting exceptions to the observations described above. Development of the coccidiostats’ chromatograms by toluene on HPTLC CN resulted in high retardation factor values. However, development by heptane on this stationary phase provided low retardation factor values, except for robenidine. Heptane can be mixed with acetone due their good miscibility, so there is the possibility of using a system with this stationary phase in the SFPE procedure.

Interesting results were also obtained with the development of chromatograms by heptane on HTPLC RP-18. Every coccidiostat, with the exception of lasalocid, was located in front of the mobile phase after the development of the chromatogram. However, the development of the chromatogram by toluene resulted in very low retention factor values, as could be predicted, so mixing of this solvent with acetone could result in obtaining an effective mobile phase in systems with HPTLC RP-18 as a stationary phase.

For further research studies on the dependence between the retention of substances and the composition of the mobile phase, methanol was used as a polar solvent for the mixture, and toluene was used as a non-polar solvent due to their very good miscibility and the lower volatility of methanol compared to acetone. To sustain scientific consistency, these two solvents were applied for the development of coccidiostats on all of the previously mentioned stationary phases.

### 2.2. Dependence of Retention of Substances vs. Composition of the Mobile Phase

**Figure 1:**
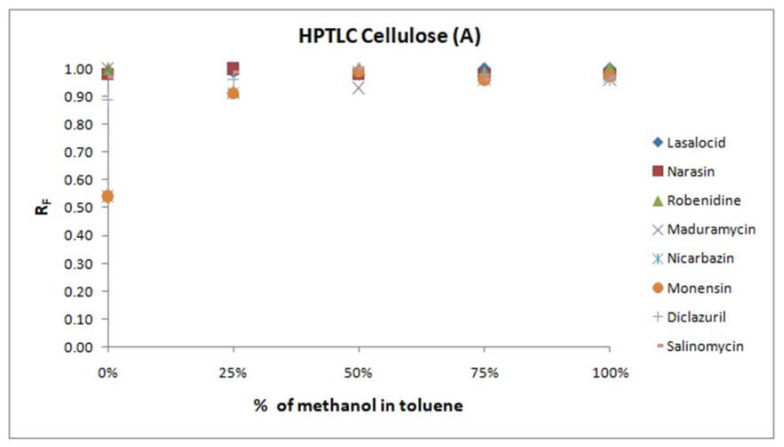

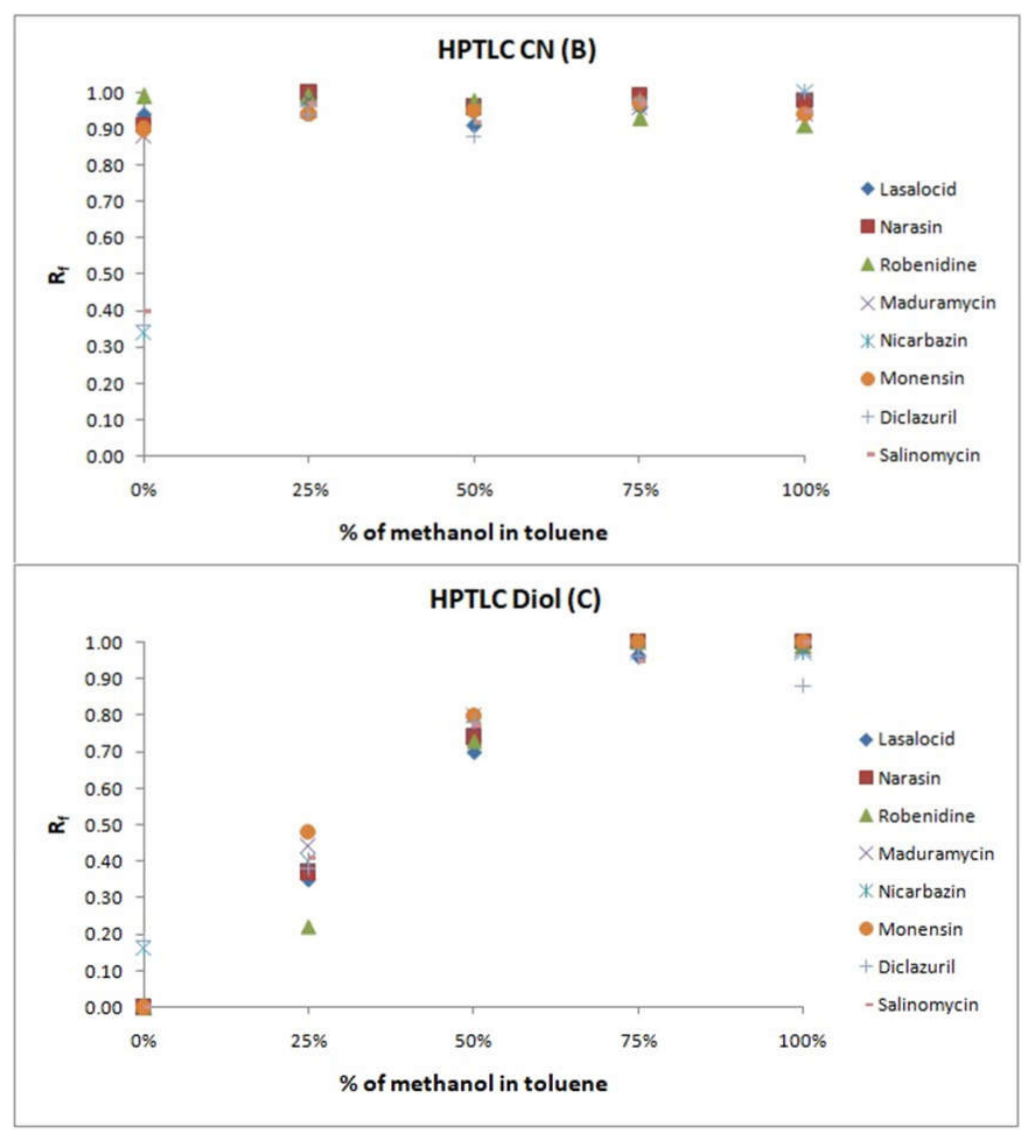

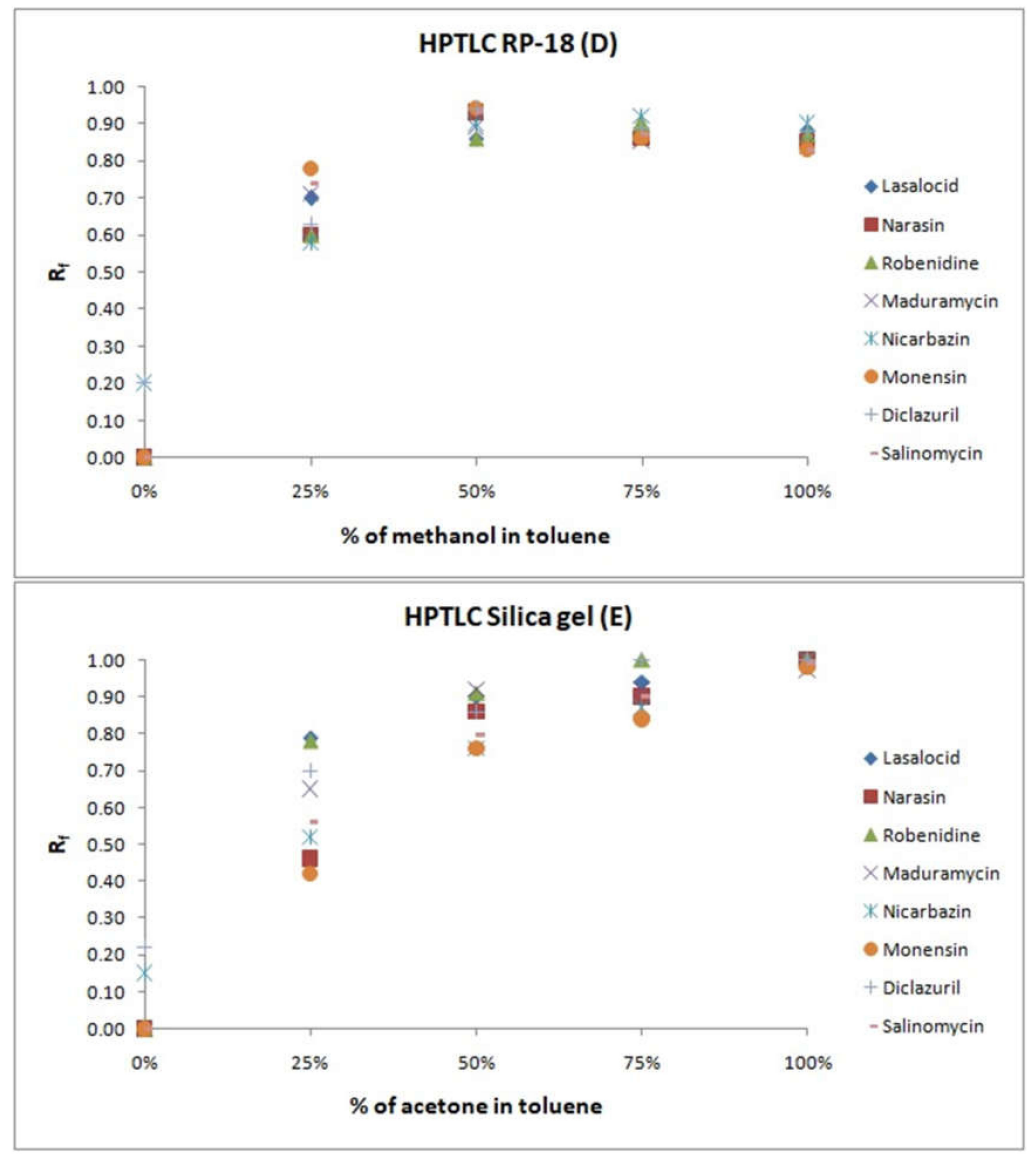

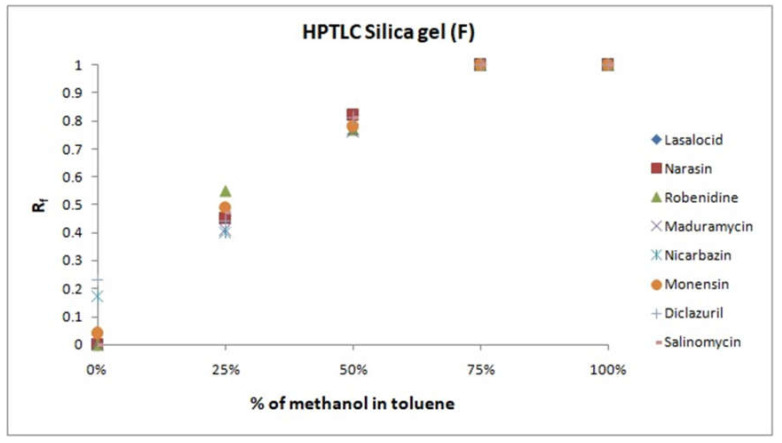
Plots of relationships of substance retention vs. composition of the mobile phase (**A**–**F**). Stationary phases are described in the headers of the plots, and mobile phases under the x-axes.

The obtained results are presented in Figure 1.

As can be noticed from Figure 1, increasing the volume fraction of methanol in the toluene solution made it possible to obtain mobile phases with increasing elution strength, which potentially allows the use of these stationary phases with methanol and toluene as mobile phases in the SFPE procedure. There were two exceptions to this observation: when HPTLC CN and HPTLC Cellulose were tested, coccidiostats’ retardation factors were close to 1, regardless of the composition of the mobile phase, which excluded the use of a mixture of methanol and toluene on these stationary phases in the SFPE procedure.

As mentioned previously, it was expected that the chromatographic system would allow the separation of substances of interest and internal standards from the matrix elements with higher and lower adsorption energy. For this purpose, substances of interest should, after the first step of the procedure, achieve a value of retardation factor of about 0.5, if possible. Based on Figure 1, this requirement can be fulfilled by HPTLC Diol with solvents of 25% to 50% methanol in toluene (*v*/*v*), HPTLC RP-18 with solvents of 20% to 40% methanol in toluene (*v*/*v*), HPTLC Silica gel solvents of 25% to 50% methanol in toluene (*v*/*v*) and with solvents of 20% to 40% acetone in toluene (*v*/*v*).

It is favorable for the results of quantitative determination when coccidiostats form a uniform dense zone on the chromatographic plate, so their retardation factors should, where possible, be similar. This fact excludes the use of HPTLC Silica gel with acetone and toluene. Moreover, the sample preparation method should, where possible, be cheap, which suggested HPTLC Silica gel with methanol and toluene as the best for further research.

For the first step of the procedure, methanol–toluene mixture in a 1:1 volume ratio as a mobile phase was established for use, because it allowed the separation of substances with lower adsorption energy from the analyzed coccidiostats, and it had a better potential to form dense zones of substances of interest than methanol–toluene mixture in a 1:3 ratio. For the second, final stage of procedure, methanol was used, because after development of the chromatogram, the target compounds and the internal standard form a single zone at the front of the solvent.

### 2.3. Application of the SFPE Procedure to Prepare Biological Samples (Premixtures) for Instrumental Analysis

The newly established conditions for the development chromatograms of coccidiostats (Section 2.2) were applied for the isolation of monensin from premixture extracts (biological samples). Moreover, this was the first time that a semi-automatic device was used for the development of the coccidiostats’ chromatograms.

**Figure 2:**
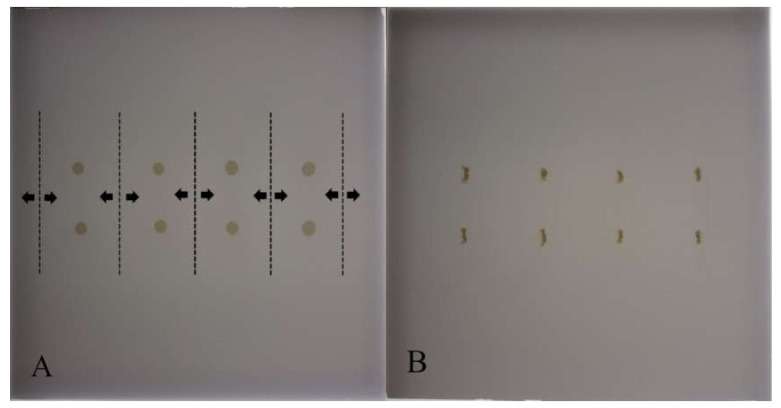
Chromatographic plate with (**A**) starting spots of the samples before their narrowing, and (**B**) starting spots of the samples after their narrowing. The dashed lines mark the path of the pipette delivering the eluent, the arrows indicate the directions of the eluent migration. Stationary phase: HPTLC Silica gel. Plates’ images were captured under white light illumination. Samples: extract from premixture with monensin.

In the first stage of SFPE, the sample zones were narrowed with methanol to facilitate the extraction of the substances from the surface of the sorbent [12], which presents in Figure 2. The smaller the surface of the substances of interest, the greater the amount of substances of interest that can be extracted, which impacts positively the precision and accuracy of the results [13,15].

**Figure 3:**
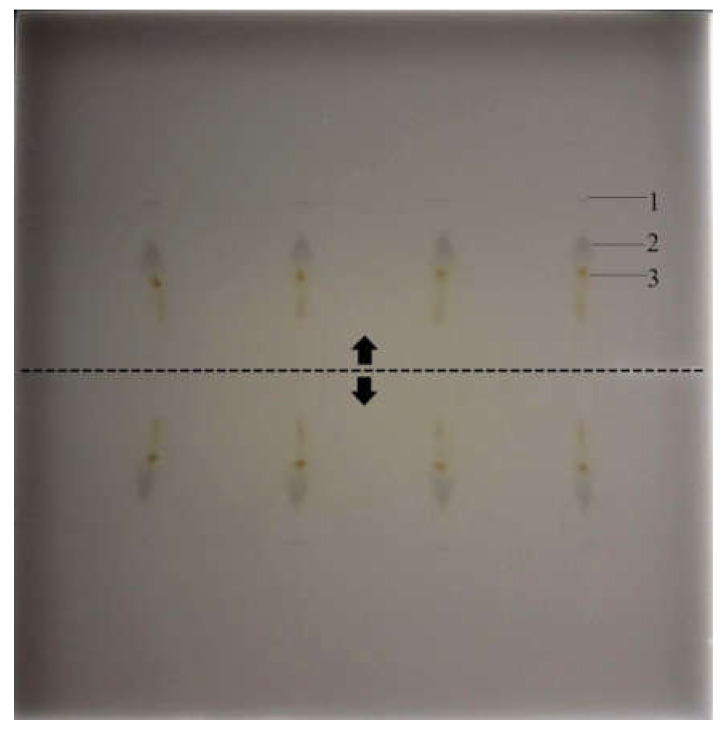
The chromatograms of the samples after the first development. The dashed line marks the path of the pipette delivering the eluent, and the arrows indicate the direction of the eluent migration. (**1**) the low retention matrix components, (**2**) the zone of the substance of interest (monensin) and the internal standard (nigericin), (**3**) matrix components of higher retention than the substances of interest. Mobile phase: 1:1 methanol–toluene (*v*/*v*). Stationary phase: HPTLC Silica gel. Plates’ images were captured under white light illumination. Samples: extract from premixture with monensin.

Then, the chromatograms of the samples were developed with a mixture of toluene and methanol in a volume ratio of 1:1 (*v*/*v*). The pipette delivering the developing solvent was moved between two rows of the samples. The supplied solvent wetted the adsorbent layer in two opposite directions, which allowed for the simultaneous development of chromatograms for double the number of samples. The chromatograms are shown in Figure 3. Simultaneous two-directional development is an indisputable novelty among methods that use planar chromatography for sample preparation. Further improvement of the procedure could make it possible to increase the number of simultaneously developed chromatograms.

**Figure 4:**
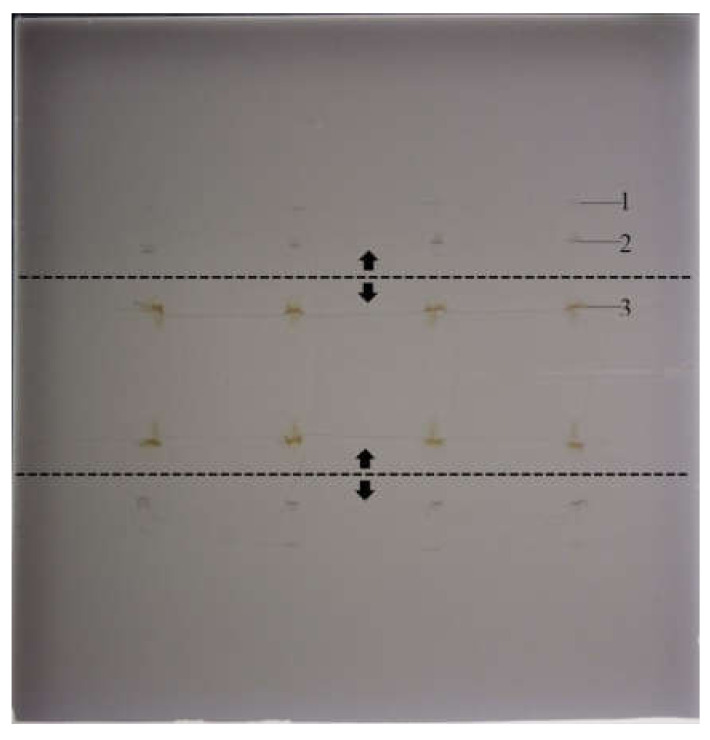
The chromatogram after focusing the substances of interest in the solvent front position. The dashed line marks the path of the pipette delivering the eluent, the arrows indicate the directions of the eluent migration. (**1**) The low retention matrix components, (**2**) the zone of the substance of interest (monensin) and the internal standard (nigericin), (**3**) high-retention matrix components. Stationary phase: HPTLC Silica gel. Plate image was captured under white light illumination. Samples: extract from premixture with monensin.

**Figure 5:**
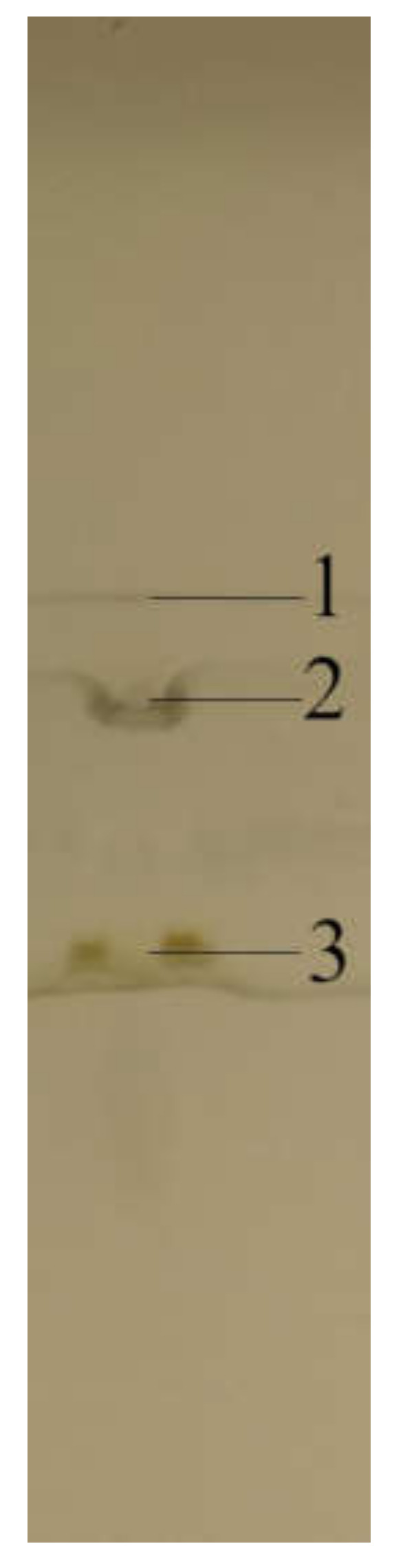
The track of chromatogram after focusing the substance of interest (salinomycin) and the internal standard (nigericin) at the solvent front position. (**1**) Low-retention matrix components, (**2**) the zone of substance of interest (salinomycin) and the internal standard (nigericin), (**3**) high-retention matrix components. Stationary phase: HPTLC Silica gel. Plate image was captured under white light illumination.

During the next stage of the procedure, the zones of the substances of interest were focused at the solvent front position (methanol). As can be observed in Figure 4 and Figure 5, they are clearly visible and easy to notice, which is undoubtedly a merit of SFPE. It is worth mentioning that the application of the semi-automatic device allowed not only for multidimensional development, but also effective separation of the substance of interest from matrix elements with high retention by starting the second stage of the procedure by developing chromatograms between the above-mentioned matrix elements and the substances of interest.

Subsequently, the substances (monensin and nigerycin as internal standard) were able to be extracted from the front position of the solvent with methanol using the TLC-MS Interface and subjected to LC-MS/MS analysis.

### 2.4. Results of Quantitative Determinations

The first step in the quantitative determination of coccidiostats was the preparation of calibration curves. LC-MS/MS made it possible to obtain peak signals, which could be compared and presented using linear regression equations (Table 2).

The calibration curves for every coccidiostat have correlation coefficients close to 1, which indicates the high effectiveness and robustness of the method.

The next step included the testing of samples of premixtures with monensin and salinomycin. The results are presented in Table 3.

Relative standard deviation values below 10% suggest that the method is precise. In comparison to previous work, in which the %RSD of the results of determination of the coccidiostats in the biological matrix ranged between 2.06 and values as high as 33.52 [15], these are satisfactory results.

Based on the data presented previously, it was possible to calculate the mass percentage of monensin and salinomycin in the tested premixtures. For monensin, it was 21.44%, and for salinomycin, 13.18%. The declared mass percentage of monensin in the premixture is minimum 19.4% and salinomycin ranges between 12.0 and 13.2%. The compliance of the results of the determination with the declared range of coccidiostat content in the premixes by manufacturers shows the reliability of the method.

## 3. Experimental

### 3.1. Materials and Reagents

Coccidiostats: diclazuril, monensin and salinomycin were kindly donated by Huvepharma Polska Sp. z o. o. Lasalocid, maduramycin, narasin, nicarbazin and robenidine were purchased from Sigma-Aldrich (St. Louis, MO, USA). Glass chromatography plates: HPTLC Cellulose F, HPTLC CN F_254_, HPTLC Diol F_254_, HPTLC RP-18 F_254_ and HPTLC Silica gel 60 F_254_ were supplied by Merck (Darmstadt, Germany). Premixtures were kindly shared by National Laboratory for Feedingstuffs in Lublin. Solvents: acetone, acetonitrile, heptane, methanol and toluene were provided by POCH (Gliwice, Poland).

### 3.2. Devices and Instrumentation

The following were used in this research: a device for cutting chromatographic plates (CAMAG, Muttenz, Switzerland), a graduated automatic pipette (Pipetman, Gilson Company, Inc., Lewis Center, OH, USA), a horizontal DS chamber for thin-layer chromatography on 10 cm × 10 cm chromatographic plates (Chromdes, Lublin, Poland), a CAMAG TLC-MS Interface device for the extraction of substances of interest from the surface of the sorbent, a CAMAG TLC Visualizer for the detection and registration of substance zones on the chromatography plate (CAMAG, Muttenz, Switzerland), a computer for operating CAMAG TLC Visualizer with WinCATS software (WinCATS-4, CAMAG, Muttenz, Switzerland), a laboratory dryer Pol-Eko 115 SLW. 21 (Pol-Eko-Aparatura, Wodzisław Śląski, Poland)), an analytical balance WPA 60/K, class I (RAD WAG, Radom, Poland), a prototype of a semiautomatic device with a moving pipette [12] for delivering the eluent to the chromatography plate (Department of Physical Chemistry, Lublin, Poland).

### 3.3. Preparation of Solutions for Qualitative Determination

The stock solutions for qualitative research were prepared by weighing the proper amount of each substance in methanol, dissolving in a suitable solvent and storing in refrigerator at 4 °C. The final concentrations of the substances, along with the solvents used, were: diclazuril: 0.0095 g/mL DMSO; lasalocid A (sodium salt): 15 μg/(0.5 mL methanol + 150 μL acetonitrile); maduramycin ammonium: 0.014 g/mL methanol; monensin sodium: 0.0378 g/mL methanol; narasin: 0.0125 g/mL methanol; nicarbazin: 0.003 g/mL DMSO; robenidine hydrochloride: 0.0034 g/mL methanol; salinomycin sodium: 0.0147 g/mL methanol.

### 3.4. Preparation of Solutions for Quantitative Determination

European Union legislation establishes ML (Maximum Level)—the maximal values of concentration of coccidiostats permitted in feed due to the unavoidable carryover of coccidiostats from target feed to non-target feeds—in order to protect sensitive animals [16]. Due this fact, these values were chosen as the reference.

Nigericin was used as the IS internal standard due to its lack of authorization as a feed additive and its similarity to the rest of the ionophores.

For each concentration level, results were obtained three times in order to calculate the average.

Concentration of solutions for quantitative determination including calibration curve: lasalocid—1.25 × 10^−5^ g/mL, 5 × 10^−6^ g/mL, 2.5 × 10^−6^ g/mL, 1.875 × 10^−6^ g/mL, 1.25 × 10^−6^ g/mL (ML), 6.25 × 10^−7^ g/mL, 2.5 × 10^−7^ g/mL; maduramycin—5 × 10^−7^ g/mL, 2 × 10^−7^ g/mL, 10^−7^ g/mL, 7.5 × 10^−8^ g/mL, 5 × 10^−8^ g/mL (ML), 2.5 × 10^−8^ g/mL, 10^−8^ g/mL; monensin—1.25 × 10^−5^ g/mL, 5 × 10^−6^ g/mL, 2.5 × 10^−6^ g/mL, 1.875 × 10^−6^ g/mL, 1.25 × 10^−6^ g/mL (ML), 6.25 × 10^−7^ g/mL, 2.5 × 10^−7^ g/mL; narasin—7 × 10^−6^ g/mL, 2.8 × 10^−6^ g/mL, 1.4 × 10^−6^ g/mL, 1.05 × 10^−6^ g/mL, 7 × 10^−7^ g/mL (ML), 3.5 × 10^−7^ g/mL, 1.4 × 10^−7^ g/mL; nigericin (IS)—10^−7^ g/mL (have not ML [7]); robenidine—7 × 10^−6^ g/mL, 2.8 × 10^−6^ g/mL, 1.4 × 10^−6^ g/mL, 1.05 × 10^−6^ g/mL, 7 × 10^−7^g/mL (ML), 3.5 × 10^−7^ g/mL, 1.4 × 10^−7^ g/mL; salinomycin—7 × 10^−6^ g/mL, 2.8 × 10^−6^ g/mL, 1.4 × 10^−6^ g/mL, 1,05 × 10^−6^ g/mL, 7 × 10^−7^g/mL (ML), 3.5 × 10^−7^ g/mL, 1.4 × 10^−7^ g/mL.

### 3.5. Preparation of the Premixture Sample Solution

First, 3 g of the premixture with monensin was weighed and transferred quantitatively into a 10 mL flask. Then, 100 μL of the internal standard solution was added. After evaporation of solvent, about 7 mL of methanol was added to flask. The suspension was shaken in an ultrasonic shaker for about 15 min, then the flask was filled up to the mark with methanol. The solution was filtered through filter paper. Then, 10 μL of the prepared solution was taken into an Eppendorf tube, and diluted with 990 μL of methanol. Then 10 μL of this solution and 990 μL of methanol was mixed to obtain final sample solution for volume of 1 mL.

The procedure for the extraction of salinomycin was different, due to the smaller concentrations to be detected. First, 2 g of the premixture was weighed and transferred quantitatively into a 10 mL flask. Then, 100 μL of the internal standard solution was added. After evaporation of the solvent, about 7 mL of methanol was added to the flask. The suspension was shaken in an ultrasonic shaker for about 15 min, then the flask was filled up to the mark with methanol. The solution was filtered through filter paper. Then, 10 μL of the prepared solution was taken into an Eppendorf tube and diluted with 1990 μL of methanol. Then 10 μL of this solution and 990 μL of methanol was mixed to obtain final sample solution for volume of 1 mL.

### 3.6. Preparation of Chromatographic Plates

Chromatographic plates were cut to the appropriate dimensions (5 cm × 10 cm or 10 cm × 10 cm) using a CAMAG device. Then they were washed with methanol and dried at 100–105 °C for 15 min. The plates were ready for use after drying and cooling.

### 3.7. Application of Samples

Small (1.5 μL) volumes of the samples were applied by an automatic pipette on the chromatographic plates.

### 3.8. Development of Chromatograms

Chromatograms were developed using a semi-automatic device using the chosen mobile phase after complete evaporation of the sample solvent from the application site. After development of the chromatogram, the mobile phase was evaporated under a fume hood.

### 3.9. Visualization of Substance Zones

There were chromatographic plates with the F_254_ factor used, which enabled observation of the substances’ zones with 254 nm or white light illumination with CAMAG TLC Visualizer. Plate images were captured with the TLC Visualizer (CAMAG).

### 3.10. Extraction of the Substances of Interest

A CAMAG TLC-MS Interface device was used for the extraction of substances of interest from the surface of the sorbent.

### 3.11. Conditions of LC-MS/MS Assay

The Agilent 1290 Infinity LC System (Santa Clara, CA, USA) connected with Agilent 6460 Triple Quadrupole was used for the LC-MS experiments. Chromatography was performed using a Zorbax Eclipse Plus C18 column (4.6 × 100 mm, 3.5 μm). The mobile phase consisted of: A: 0.1% formic acid in water; and B: 0.1% formic acid in methanol. The gradient elution was conducted as follows: 0 min: 95% A, 5% B; 1 min: 95% A, 5% B; 3 min: 5% A, 95% B; 11 min: 5% A, 95% B; 11.50 min: 95% A, 5% B; 14 min: 95% A, 5% B. 5 min: 50% A, 50% B; 5.1 min: 5% A, 95% B. The extracting mobile phase was comprised of pure methanol; flow rate: 0.4 mL/min. MS data were acquired in the positive ion modes (multiple-reaction monitoring mode) with electrospray probe voltages of 3500 V. The nebulizer gas setting was 40 psi. The ion source was operated at a temperature of 300 °C and a drying gas setting of 7 L/min. Transition MRM: lasalocid—613.4 (primary ion), 377.3 and 595.3 (secondary ions); maduramycin—934.9 (primary ion), 629.3 and 647.3 (secondary ions); monensin—693.3 (primary ion), 675.3 and 461.1 (secondary ions); narasin—787.5 (primary ion), 431.2 and 531.3 (secondary ions), nigericin—747.4 (primary ion), 703.3 and 501.23 (secondary ions); robenidine—334.0 (primary ion), 138.0 and 154.9 (secondary ions); salinomycin—773.4 (primary ion), 531.1 and 431.1 (secondary ions).

## 4. Conclusions

The SFPE procedure made it possible to separate substances of interest from matrix components with both lower and higher retention from the analytes, as shown in this report. Different adsorbents and mobile phases were used to prepare the samples, enabling the selection of the satisfactory chromatographic systems depending on the properties of the substances of interest. Systems were found for the eight selected coccidiostats.

Based on the presented observations and research results, it was also concluded that the type of stationary phase used, as well as the mobile phase used, affects the retention and selectivity of the separation of the substances of interest.

This is the first time that real market samples have been investigated using the SFPE technique. The conditions applied for the development were effective for the isolation of monensin and salinomycin from biological matrix. It can be assumed that its usage with other premixtures could also be useful. These conditions are relatively simple to apply, and require simple and cheap materials, such as HPTLC Silica gel plates, methanol and toluene, which is undoubtedly an advantage of the method.

Introducing the semi-automatic device was highly advantageous for the research. It allowed the simultaneous chromatogram development of many samples, which indicated its further usefulness for the next investigations.

The quantitative results obtained were precise, indicating progress when compared to previous research. The reliability of the results showed the great capability of SFPE method for application in the preparation of samples with coccidiostats. The method has great potential for routine testing of various biological samples for the presence of coccidiostats. In the future, it could be successfully used to prepare samples of other substances of interest.

## Figures and Tables

**Table 1:** Values of R_f_ coefficient of coccidiostats for tested chromatographic systems.

Stationary Phase	HPTLC Cellulose								HPTLC CN							
**Mobile Phase**	**Lasalocid**	**Narasin**	**Robenidine**	**Maduramycin**	**Nicarbasin**	**Monensin**	**Diclazuril**	**Salinomycin**	**Lasalocid**	**Narasin**	**Robenidine**	**Maduramycin**	**Nicarbasin**	**Monensin**	**Diclazuril**	**Salinomycin**
**Toluene**	0.99	0.98	1.00	1.00	0.54	0.97	0.89	0.97	0.94	0.91	0.99	0.88	0.34	0.90	0.36	0.40
**Methanol**	1.00	0.98	1.00	0.96	0.97	0.96	0.96	0.97	0.98	0.98	0.91	0.94	1.00	0.94	1.00	0.95
**Acetone**	0.98	0.98	0.98	0.99	1.00	0.98	1.00	0.98	1.00	1.00	1.00	0.97	1.00	0.98	0.96	0.96
**Heptane**	0.41	1.00	0.00	1.00	0.00	0.00	0.00	0.00	0.00	0.00	0.58	0.18	0.00	0.15	0.00	0.10
**Acetonitrile**	0.98	1.00	0.97	1.00	1.00	1.00	1.00	0.95	0.99	0.98	0.95	1.00	1.00	0.95	1.00	1.00
**Stationary Phase**	**HPTLC Diol**								**HPTLC RP-18**							
**Mobile Phase**	**Lasalocid**	**Narasin**	**Robenidine**	**Maduramycin**	**Nicarbasin**	**Monensin**	**Diclazuril**	**Salinomycin**	**Lasalocid**	**Narasin**	**Robenidine**	**Maduramycin**	**Nicarbasin**	**Monensin**	**Diclazuril**	**Salinomycin**
**Toluene**	0.00	0.00	0.00	0.00	0.16	0.00	0.18	0.00	0.00	0.00	0.00	0.00	0.20	0.00	0.20	0.00
**Methanol**	1.00	1.00	0.99	0.98	0.97	1.00	0.88	1.00	0.88	0.85	0.87	0.84	0.90	0.83	0.88	0.83
**Acetone**	0.98	1.00	1.00	0.97	0.97	0.95	0.98	0.98	0.98	1.00	0.89	0.96	0.97	1.00	0.97	1.00
**Heptane**	0.00	0.00	0.00	0.00	0.02	0.00	0.00	0.00	0.38	1.00	1.00	1.00	1.00	1.00	1.00	1.00
**Acetonitrile**	1.00	1.00	0.98	1.00	0.96	1.00	0.99	1.00	0.94	0.87	0.78	0.80	0.91	0.98	0.98	0.97
**Stationary Phase**	**HPTLC Silica Gel**															
**Mobile Phase**	**Lasalocid**	**Narasin**	**Robenidine**	**Maduramycin**	**Nicarbasin**	**Monensin**	**Diclazuril**	**Salinomycin**								
**Toluene**	0.00	0.00	0.00	0.00	0.15	0.00	0.22	0.00								
**Methanol**	1.00	1.00	1.00	1.00	1.00	1.00	1.00	1.00								
**Acetone**	1.00	1.00	1.00	0.97	1.00	0.98	1.00	0.99								
**Heptane**	0.00	0.00	0.00	0.00	0.00	0.00	0.00	0.00								
**Acetonitrile**	0.60	0.52	1.00	0.78	0.99	0.54	0.85	0.57								

**Table 2:** Linear regression equations for the relationships between substance of interest/nigericin peak area ratios and substance of interest concentrations.

Substance of Interest	Linearity	R^2^
Lasalocid	y = 2.868x − 1.139	0.980
Maduramycin	y = 0.022x + 0.032	0.981
Monensin	y = 17.02x	0.990
Narasin	y = 4.039x + 0.911	0.994
Robenidine	y = 3.192x + 0.988	0.990
Salinomycin	y = 0.882x + 0.227	0.999

**Table 3:** Results of quantification of monensin and salinomycin in extracts from premixtures. Number of samples = 8.

Substance of Interest in Premixture	Percentage of Substance of Interest in Premixture	Standard Deviation	%RSD
Monensin	21.44%	7.725886	8.63%
Salinomycin	13.18%	0.155344	8.29%
